# Multiplexed multimodal single-cell technologies: From observation to perturbation analysis

**DOI:** 10.1016/j.mocell.2024.100147

**Published:** 2024-11-08

**Authors:** Su-Hyeon Lee, Junha Park, Byungjin Hwang

**Affiliations:** 1 Department of Biomedical Sciences, Yonsei University College of Medicine, Seoul, South Korea; 2 Brain Korea 21 Project, Graduate School of Medical Science, Yonsei University College of Medicine, Seoul, South Korea; 3 Yonsei University College of Medicine, Seoul, South Korea

**Keywords:** Combinatorial indexing, CRISPR, Multiomics, Single-cell sequencing

## Abstract

Single-cell technologies have undergone a significant transformation, expanding from their initial focus on transcriptomics to encompass a diverse range of modalities. Recent advancements have markedly improved scalability and reduced costs, facilitating the processing of larger cell populations and broadening the scope of single-cell research. The incorporation of clustered regularly interspaced short palindromic repeats (CRISPR)-based perturbations has revolutionized the field by enabling precise functional genomics and detailed studies of gene regulation at the single-cell level. Despite these advancements, challenges persist, particularly in achieving genome-wide perturbations and managing the complexity of high-throughput data. This review discusses the technological milestones that have driven these changes, the current limitations of single-cell CRISPR technologies, and the future directions needed to address these challenges and advance our understanding of cellular biology.

## INTRODUCTION

The field of single-cell transcriptomics has evolved significantly since the concept of single-cell transcriptome analysis was first established (Tang et al., 2009). A major milestone was achieved 2 years later with the introduction of barcoding techniques for multicellular analysis, which accelerated the advancement of single-cell technology (Islam et al., 2011).

**Fig. 1:**
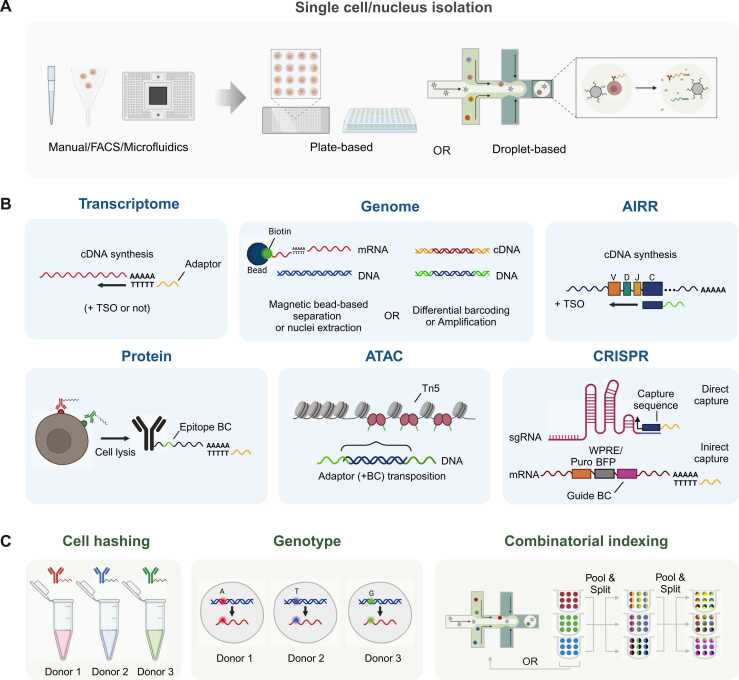
Overview of single-cell omics technologies. (A) Single-cell isolation methods, where cells are processed using plate-based or droplet-based systems. (B) Various modalities available for single-cell multiomics analysis, with a focus on chemistries that integrate transcriptomics. (C) Scalable technologies are designed to increase the throughput and processing capacity of single-cell analysis, enabling the simultaneous study of larger cell populations across diverse modalities. AIRR, adaptive immune receptor repertoire; sgRNA, single guide RNA.

The single-cell–level enzymatic reactions, enabled by innovations in microplates, microfluidic chambers, and droplet-based systems, have expanded the technology beyond transcriptomics. It now includes genome and epigenome analysis, epitope profiling, and adaptive immune receptor repertoire (AIRR) sequencing (Buenrostro et al., 2015, Dey et al., 2015, Stoeckius et al., 2017, Tu et al., 2019). This innovation has been accompanied by an exponential increase in the scale of analyses, progressing from dozens of cells to millions over the past decade (Jia et al., 2022). The introduction of clustered regularly interspaced short palindromic repeats (CRISPR) genome editing technology has accelerated the integration of functional genomics with single-cell analysis (Jinek et al., 2012). This has led to the development of CRISPR-based perturbations combined with various single-cell technologies, enabling the digital tracking of gene modifications and their effects on cellular behavior. Perturb-seq and CRISPR droplet sequencing (CROP-seq) have emerged, facilitating the systematic study of gene function by linking genetic perturbations to transcriptional responses in single cells (Datlinger et al., 2017, Dixit et al., 2016) (Table 1, Fig. 1).

**Table 1:** Recent advancements in single-cell multiomics technologies

Year	Method	Cell isolation	Modality	Throughput	Per experiment	References
2015	DR-seq	Plate-based; manual	DNA, mRNA	Low	∼50	Dey et al. (2015)
	G&T-seq	Plate-based; FACS	DNA, mRNA	Low	∼200	Macaulay et al. (2015)
	Drop-seq	Microfluidics droplet	mRNA	High	∼100k	Macosko et al. (2015)
	InDrop	Microfluidics droplet	mRNA	High	∼10k	Klein et al. (2015)
	scATAC	Microfluidics chip	Chromatin accessibility	Medium	∼1k	Buenrostro et al. (2015)
	scCHIP-seq	Microfluidics droplet	Chromatin-protein complex, mRNA	High	∼10k	Rotem et al. (2015)
	scHi-C	Plate-based; manual	DNA conformation	N/A	N/A	Nagano et al. (2015)
2016	scTrio-seq	Plate-based; manual	DNA, mRNA, DNA methylation	Low	∼50	Hou et al. (2016)
	Perturb-seq	Microfluidics droplet (Chromium)	mRNA, sgRNA (indirect)	High	∼100k	Dixit et al. (2016)
	CRISP-seq	Plate-based; FACS	mRNA, sgRNA (indirect)	Medium	∼1k	Jaitin et al. (2016)
2017	CITE-seq	Microfluidics droplet (Chromium)	mRNA, surface protein	High	∼100k	Stoeckius et al. (2017)
	REAP-seq	Microfluidics droplet (Chromium)	mRNA, surface protein	High	∼100k	Peterson et al. (2017)
	sci-RNA-seq	Plate-based	mRNA	Ultra-high	∼400k	Cao et al. (2017)
	CROP-seq	Microfluidics droplet	mRNA, sgRNA (indirect)	High	10k-50k	Datlinger et al. (2017)
	Mosaic-seq	Microfluidics droplet	mRNA, sgRNA (indirect)	High	∼10k	Xie et al. (2017)
2018	SIDR	Plate-based	Nucleic DNA, cytosolic mRNA	Low	∼50	Han et al. (2018)
	sci-CAR	Plate-based	mRNA, chromatin accessibility	High	∼10k	Cao et al. (2018)
	scNMT-seq	Plate-based; FACS	mRNA, DNA methylation, chromatin accessibility	Low	∼200	Clark et al. (2018)
	SPLiT-seq	Plate-based	mRNA	High	100k	Rosenberg et al. (2018)
2019	SNARE-seq	Microfluidics droplet	mRNA, chromatin accessibility	High	10k	Chen et al. (2019)
	RAID-seq	Plate-based	mRNA, intracellular protein	Low	−500	Gerlach et al. (2019)
	ECCITE-seq	Microfluidics droplet	mRNA, surface protein, sgRNA (direct)	High	∼100k	Mimitou et al. (2019)
	sci-RNA-seq 3	Plate-based	mRNA	Ultra-high	Over 2 mil	Cao et al. (2019)
	dsciATAC	Microfluidics droplet	Chromatin accessibility	High	∼100k	Lareau et al. (2019)
	Paired-seq	Plate-based	mRNA, chromatin accessibility	Ultra-high	Over 1 mil	Zhu et al. (2019)
	Perturb-ATAC-seq	Microfluidics chip	Chromatin accessibility, sgRNA (direct)	Medium	∼5k	Adam Rubin et al. (2019)
	crisprQTL	Microfluidics droplet	mRNA, sgRNA (indirect)	High	∼100k	Gasperini et al. (2019)
2020	TARGET-seq	Plate-based; FACS	Targeted DNA, targeted mRNA	Medium	∼1k	Rodriguez-Meira et al. (2020)
	SHARE-seq	Plate-based	mRNA, chromatin accessibility	High	∼100k	Ma et al. (2020)
	Direct-capture Perturb-seq	Microfluidics droplet (Chromium)	mRNA, sgRNA (direct)	High	∼100k	Replogle et al. (2020)
	Direct-seq	Microfluidics droplet/chip	mRNA, sgRNA (direct)	Low	∼100	Song et al. (2020)
	TAP-seq	Microfluidics droplet (Chromium)	mRNA, sgRNA (indirect)	High	∼100k	Schraivogel et al. (2020)
	POKI-seq	Microfluidics droplet (Chromium)	mRNA, HDR	High	∼100k	Roth et al. (2020)
2021	inCITE-seq	Microfluidics droplet (Chromium)	mRNA, intracellular protein	High	∼100k	Chung et al. (2021)
	ASAP-seq	Microfluidics droplet (Chromium)	Surface protein, chromatin accessibility	High	∼100k	Mimitou et al. (2021)
	scifi-RNA-seq	Microfluidics droplet	mRNA	Ultra-high	Over 1 mil	Datlinger et al. (2021)
	SCITO-seq	Microfluidics droplet	mRNA, surface protein	Ultra-high	Over 1 mil	Hwang et al. (2021)
	sc-Tiling	Microfluidics droplet (Chromium)	mRNA, sgRNA (direct)	High	∼100k	Yang et al. (2021)
	CRISPR-sciATAC	Plate-based	Chromatin accessibility, sgRNA (indirect)	High	∼30k	Liscovitch-Brauer et al. (2021)
	Spear-ATAC	Microfluidics droplet (Chromium)	Chromatin accessibility, sgRNA (direct)	High	∼100k	Pierce et al. (2021)
	Perturb-CITE-seq	Microfluidics droplet (Chromium)	mRNA, surface protein, sgRNA (indirect)	High	∼100k	Frangieh et al. (2021)
2022	sc-eVIP	Microfluidics droplet (Chromium)	mRNA, coding variants (indirect)	High	∼100k	Ursu et al. (2022)
	scPCOR-seq	Plate-based	Chromatin-protein complex, mRNA	High	∼10k	Pan et al. (2022)
2023	FIPRESCI	Microfluidics droplet (Chromium)	mRNA, AIRR	Ultra-high	Over 1 mil	Li et al. (2023)
	TISCC-seq	Microfluidics droplet (Chromium)	mRNA, target script	High	∼100k	Kim et al. (2023)
2024	SUM-seq	Microfluidics droplet (Chromium)	mRNA, chromatin accessibility	Ultra-high	Over 1 mil	Lobato-Moreno et al. (2024)
	scifi-ATAC-seq	Microfluidics droplet (Chromium)	Chromatin accessibility	Ultra-high	Over 1 mil	Zhang et al. (2024)
	txci-ATAC-seq	Microfluidics droplet (Chromium)	Chromatin accessibility	Ultra-high	Over 1 mil	Zhang et al. (2024)
	NSC-seq	Microfluidics droplet	mRNA, sgRNA (direct)	N/A	N/A	Islam et al. (2024)

Single-cell omics technologies are categorized by publication year. It includes the technology name, single-cell isolation method, modality, and cell throughput. For droplet-based technologies, throughput is based on the maximum number of droplets generated per microfluidic run (eg, 8 channels for Chromium). Throughput is also categorized as low (<1k), medium (<10k), high (<200k), or ultra-high (>200k). Entries marked as N/A indicate that data are not available.

AIRR, adaptive immune receptor repertoire; sgRNA, single guide RNA.

This review will explore the evolution of single-cell omics technologies from various perspectives, including modality and throughput. We will also discuss the significance of integrating these technologies with functional genomics and address the current limitations and challenges in the field.

## SINGLE-CELL MULTIOMICS TECHNOLOGIES

The pursuit of extracting multilayered information from single cells began with advances in combining transcriptome and genome analyses. The first-generation single-cell multiomics technologies, such as gDNA-mRNA sequencing (DR-seq) and genome-and-transcriptome sequencing (G&T-seq), marked significant progress by enabling the simultaneous observation of gene expression and genome-wide copy numbers within individual cells (Dey et al., 2015, Macaulay et al., 2015). Despite subsequent innovations such as simultaneous isolation of genomic DNA and total RNA (SIDR) and TARGET-seq, there are still important considerations associated with whole genome sequencing, including high costs per cell and allelic or locus dropout (Han et al., 2018, Rodriguez-Meira et al., 2020). In light of these considerations, there is growing attention to transcriptomic analyses that account for allele-specific expression and coding mutations, which can enhance the integration of genome and transcriptome data (Deng et al., 2014, Muyas et al., 2023, Petti et al., 2019, Qi et al., 2023).

Concurrently, multiomics approaches have emerged that leverage short-read sequencing technologies, barcoding for indirect modality information, and the efficient cell processing capabilities of microfluidic droplet platforms (Buenrostro et al., 2015, Stoeckius et al., 2017). Epigenome analysis has gained significant attention for its ability to provide valuable insights into the upstream regulatory mechanisms of the transcriptome in individual cells. This modality leverages a robust technical foundation, incorporating established methods such as assay for transposase-accessible chromatin using sequencing (ATAC-seq), chromatin immunoprecipitation sequencing (ChIP-seq), and high-throughput chromatin conformation capture (Hi-C), which facilitate detailed exploration of chromatin accessibility, protein-DNA interactions, and genome organization, respectively (Buenrostro et al., 2015, Nagano et al., 2015, Rotem et al., 2015). Consequently, epigenome analysis has become a key component of single-cell multiomics technology, offering rich, complementary data to enhance our understanding of gene expression and cellular function (Muto et al., 2021, Pan et al., 2022, Wu et al., 2024). In a recent study, single-cell RNA and ATAC analysis revealed a TIM-3+ CD8 T cell subpopulation with a terminally exhausted phenotype and critical antitumor capacity associated with BATF motif activity. This is an example of how multiomics can be used to precisely phenotype cells while simultaneously uncovering functional mechanisms (Minnie et al., 2024).

Single-cell ATAC-seq has been further integrated into various single-cell technologies (Buenrostro et al., 2015). For instance, combinatorial indexing methods such as sci-CAR and SHARE-seq (Cao et al., 2018, Ma et al., 2020), along with droplet-based techniques such as SNARE-seq (Chen et al., 2019), enable concurrent analysis of transcriptome and chromatin accessibility. The commercial adoption of these technologies by companies, such as 10X Genomics, has facilitated seamless integration of RNA and epigenome analyses. Additionally, advanced trimodal approaches, including scTrio-seq and scNMT-seq, exhibit considerable promise for a wide range of applications in single-cell multiomics (Clark et al., 2018, Hou et al., 2016).

In the realm of single-cell analysis, protein analysis has historically been at the forefront due to the long-established reputation of flow cytometry, a technique developed in the 1960s (Dittrich and Gühde, 1971). This prominence in single-cell analysis was further advanced by innovations such as cellular indexing of transcriptomes and epitopes by sequencing (CITE-seq) and RNA expression and protein sequencing assay (REAP-seq), introduced in 2017 (Peterson et al., 2017, Stoeckius et al., 2017). These methods utilize existing flow cytometry antibodies by conjugating them with oligonucleotides, which enables the conversion of protein expression data into sequence-read information. While quantification of proteins using such oligonucleotide-based methods was originally proposed with immune-PCR, its conjunction with modern paired-end sequencing techniques and droplet-based systems marks a significant advancement (Niemeyer et al., 2005). Although these methods do not represent proteome analysis, as they still depend on antibodies, they offer substantial advantages by mitigating the signal overlap issues of traditional flow cytometry, expanding the range of epitopes. The development of single-cell RNA and Immuno-detection (RAID-seq) and intranuclear CITE-seq (inCITE-seq) has enabled the analysis of intracellular proteins by utilizing plate-based and droplet-based systems, further advancing the depth of protein analysis in single-cell technologies (Chung et al., 2021, Gerlach et al., 2019).

The need for simultaneous AIRR and RNA analysis arises from the necessity to understand both the functional characteristics of immune cells along with their clonotypes at the single-cell level. The widely used 10X Chromium system and many alternative technologies adopt a 5' end sequencing approach to sequence the V(D)J region via short-read sequencing (Benotmane et al., 2023). Various tools have been developed to generate dimensional reductions or shared representations that comprehensively reflect both T cell or B cell clonotype information and transcriptomic data. TCR functional landscape estimation supervised with scRNA-seq analysis (TESSA) employs a Bayesian approach to iteratively construct a TCR network and estimate the association between TCR embedding and gene expression (Zhang et al., 2021). Benisse identifies a common latent space that couples BCR embedding with gene expression and detects BCR networks within this latent space (Zhang et al., 2022). Clonotype neighbor graph analysis (CoNGA) constructs graphs for gene expression and TCR, identifying graph-graph and graph-feature similarities across modalities (Schattgen et al., 2021). Additionally, single-cell inference of class-switch recombination (sciCSR) utilizes a hidden Markov model to learn and predict B cell class switch dynamics by integrating B cell gene expression and BCR profiles (Ng et al., 2023). These tools explore the relationships between AIRR and RNA data. Integrating these data allows researchers to trace lymphocyte lineages, understand cell differentiation during clonal expansion, and predict epitopes. In 2021, a pivotal study analyzed peripheral blood mononuclear cells from over 100 COVID-19 patients across varying severities using single-cell RNA, protein, and AIRR multiomics technologies. This integrative approach, combining RNA data with surface protein expression, enabled refined cell type annotations and a more precise understanding of immune cell activation and interactions. TCR analysis further illuminated clonal expansion patterns associated with disease severity, while BCR profiling revealed gender-specific differences in clonality and mutation frequency. This research has provided a significant foundation for subsequent studies on the immune response to COVID-19 (Stephenson et al., 2021).

Single-cell multiomics technologies have evolved rapidly over a short period, with each technology adapting to advancements in others, resulting in dynamic and interdependent development. Consequently, contemporary technologies exhibit a blend of features from earlier methods, resulting in approaches that are both mutually compatible and feasible. For example, expanded CRISPR-compatible cellular indexing of transcriptomes and epitopes by sequencing (ECCITE-seq) extends the capabilities of CITE-seq by integrating cell hashing, RNA, protein, and V(D)J analysis (Mimitou et al., 2019). ATAC with select antigen profiling by sequencing (ASAP-seq) merges single-cell ATAC sequencing (scATAC-seq) with oligo-labeled antibodies to simultaneously assess proteins and chromatin accessibility (Mimitou et al., 2021). These innovative combinations represent a significant leap forward in single-cell analysis, offering new ways to explore cellular complexity and function.

## SCALABLE TECHNOLOGIES FOR SINGLE-CELL MULTIOMICS

In single-cell multiomics technologies, scalability is as crucial as the innovative approaches used for sequencing diverse modalities. This discussion assesses scalability from two key perspectives: the number of samples that can be distinguished in a single experiment and sequencing run, and the number of cells that can be processed simultaneously.

The core value of single-cell analysis is in capturing diversity within and between biological conditions, requiring concurrent analysis of multiple samples to minimize batch effects and enhance productivity. Addressing these challenges, Ye's group developed a method utilizing “natural barcodes,” such as SNPs, for demultiplexing (Kang et al., 2017). By leveraging genetic variants, this technique effectively deconvolves pooled transcriptomes. Furthermore, identifying genetic variants of different donor origins from a single droplet introduces a novel method for multiplet removal, improving upon traditional biological doublet detection. This innovation has set a new standard in computational tool development, leading to algorithms such as Souporcell, Vireo, and scSplit (Heaton et al., 2020, Huang et al., 2019, Xu et al., 2019). These tools have advanced to enable genotype-free demultiplexing, offering robust and efficient solutions for scenarios where specific donor identification is unnecessary, such as simultaneous analysis of samples from the same experimental group or when prior genotype information is unavailable.

Around the same period, the developers of CITE-seq introduced the concept of cell hashing by demonstrating that barcodes included in oligo-conjugated antibodies could be used for sample identification (Stoeckius et al., 2018). This experimental approach enables sample multiplexing and the detection of multiplets containing multiple hashtags. Despite the risk of cell loss due to nonspecific cross-binding, it is an appealing alternative when genotype-based methods are impractical, such as in studies of paired samples from the same individual. While cell hashing necessitates additional experimental steps, using genotype-based methods involves generation of genetic variant reference from BAM files and demands substantial computational resources for the deconvolution (Brouard et al., 2019). In this context, each approach consequently embodies its own distinct strengths and limitations.

With the availability of various sample multiplexing methods suited to different research needs, the ability to process large numbers of cells has directly contributed to significant cost reductions in research. The evolution of single-cell analysis technology has seen significant advancements in cell processing capabilities. Beginning with manual cell picking, the field progressed to FACS-based single-cell isolation, microwell plates, microfluidic chambers, and droplet-based systems (DeLaughter, 2018, Klein et al., 2015, Macosko et al., 2015, Tang et al., 2009, Rodriguez-Meira et al., 2020). While this increase has significantly reduced the cost per cell, challenges remain, particularly for rare cell identification, large cohort studies, and cell atlas construction. Consequently, researchers continue to focus on enhancing throughput to address these ongoing needs.

A major advancement in ultra–high-throughput single-cell sequencing is the introduction of single-cell combinatorial indexing RNA sequencing (sci-RNA-seq) (Cao et al., 2017). This technique employs a combinatorial indexing approach to analyze hundreds of thousands of cells in a single experiment. Split-pool ligation-based transcriptome sequencing (SPLiT-seq) provides ultra–high-throughput capabilities, enabling the analysis of millions of cells with standard laboratory equipment, achieving a remarkably low library construction cost (Rosenberg et al., 2018). sci-RNA-seq 3, capable of processing around 2 million cells per analysis, represents a notable advancement, though multiple rounds of indexing on plates pose feasibility challenges (Cao et al., 2019).

The introduction of single-cell combinatorial fluidic indexing (scifi)-RNA-seq 2 years later represented a major development by integrating combinatorial indexing with droplet microfluidics (Datlinger et al., 2021). By assigning indices to every single transcript, individual sequencing reads can be traced back to their cell origins even if multiplets are formed. This approach effectively addresses the major limitation of droplet-based technologies—unanalyzable multiplets—allowing for processing of over 15 times more cells than traditional methods. Building on these, droplet-based ultra–high-throughput approaches have been extended to other modalities. Droplet-based single-cell combinatorial indexing for ATAC-seq (dsciATAC) enables large-scale single-cell chromatin accessibility profiling (Lareau et al., 2019) and scifi-ATAC-seq further advanced the throughput with reduced cross-cell contamination compared to earlier methods (Zhang et al., 2024b). 10X-compatible combinatorial indexing ATAC-seq (txci-ATAC-seq) provides enhanced performance over dsciATAC, further refining single-cell chromatin accessibility analysis (Zhang et al., 2024a).

These advancements have also been applicable to multiomics technologies. Single-cell combinatorial indexed cytometry sequencing (SCITO-seq) leveraged splint oligo-tagged antibodies compatible with various 10X Genomics Chromium systems, facilitating ultra–high-throughput analysis of proteins (Hwang et al., 2021). In 2023, five prime end single-cell combinatorial indexing RNA sequencing (FIPRESCI) also combined multiple indexing rounds with Chromium’s 5' end RNA and V(D)J analysis method (Li et al., 2023). Paired-seq and single-cell ultra-high-throughput multiomic sequencing (SUM-seq) utilize combinatorial indexing to efficiently perform simultaneous RNA and ATAC analysis, with Paired-seq employing a plate-based approach and SUM-seq utilizing a droplet-based system (Lobato-Moreno et al., 2024, Zhu et al., 2019). These scalable single-cell technologies have unlocked new opportunities for extensive screening, multiplexed experiments, and the analysis of rare cell populations, placing a responsibility on researchers to leverage these advancements for detailed scientific inquiries.

## OVERVIEW OF SINGLE-CELL CRISPR TECHNOLOGIES

Although the advent of single-cell technologies has uncovered the rich tapestry of cell types and states that comprise biological systems, understanding how specific genetic factors influence these intricate cellular landscapes requires more than just observational data. Integrating functional genomics with single-cell approaches offers a transformative perspective, enabling a deeper comprehension of gene regulation at the individual cell level.

In 2016, significant progress was achieved with the introduction of Perturb-seq and CRISP-seq (Dixit et al., 2016, Jaitin et al., 2016). These pioneering studies combined single-cell and CRISPR technologies to analyze transcriptome changes resulting from genetic perturbations. Both facilitated single guide RNA (sgRNA) identification by inserting guide-identifying barcodes into the mRNA transcript, enabling the use of conventional mRNA analysis systems for sgRNA detection. In contrast, CROP-seq incorporated guide sequences into the 3′ long terminal repeat, enabling them to be transcribed alongside mRNA without interfering with transcriptional activity (Datlinger et al., 2017). This approach enabled the direct identification of gRNA sequence, eliminating the need for barcode insertion. As pioneers in this area, aforementioned methods merged the strengths of 2 traditional CRISPR screening approaches—pooled and arrayed screening—enabling efficient treatment with multiple guide RNAs while allowing detailed analysis of individual transcriptomes.

Following this, many technologies began to adopt mRNA-independent methods, utilizing invariant regions of sgRNA as annealing sites for reverse transcription. For example, ECCITE-seq, which relies on a 5′ end sequencing system, demonstrated the ability to capture sgRNA using gel bead oligos, enabling the simultaneous analysis of transcriptome, protein, V(D)J, and sgRNA (Mimitou et al., 2019). Direct-capture Perturb-seq and Direct-seq also focus on directly capturing sgRNA (Replogle et al., 2020, Song et al., 2020). Native sgRNA capture and sequencing (NSC-seq) further advanced this approach by capturing the canonical scaffold of sgRNA, allowing for the use of conventional KO libraries instead of relying on modified constant regions (Islam et al., 2024). Transcript-informed single-cell CRISPR sequencing (TISCC-seq), on the other hand, uses a completely different approach to identify perturbations. Unlike other technologies that rely on guide sequence or barcodes, TISCC-seq uses nanopore long-read sequencing to directly sequence the mutations in the target gene, still obtaining the transcriptome through short-read sequencing. This not only avoids the variant-barcode mis-association issue but also distinguishes multiple genotypes induced by a single perturbation (Kim et al., 2023).

In addition to capturing strategies, technologies tailored for specific screening applications have also been developed. For instance, targeted Perturb-seq (TAP-seq) selectively sequences target mRNA, enabling cost-effective production of high-quality data (Schraivogel et al., 2020). sc-Tiling focuses on mutagenesis within specific domains of coding regions (Yang et al., 2021), while pooled knockin sequencing (POKI-seq) is designed for large-scale knock-in screening (Roth et al., 2020). Single-cell expression-based variant impact phenotyping (sc-eVIP) integrated an open reading frame library of oncogene coding variants with corresponding barcodes into Perturb-seq, providing insights into how somatic variants drive oncogenesis (Ursu et al., 2022). Furthermore, technologies such as Perturb-ATAC, CRISPR-sciATAC, and single-cell perturbations with an accessibility read-out using scATAC-seq (Spear-ATAC) have been developed to investigate interactions between genetic variants and chromatin accessibility profiles (Adam Rubin et al., 2019, Liscovitch-Brauer et al., 2021, Pierce et al., 2021). Mosaic-seq combines dCas9 with enhancer repressors to screen enhancer activity (Xie et al., 2017) and crisprQTL has been advanced with improved template switching rates and multiplicity of infection (Gasperini et al., 2019). This ongoing evolution of single-cell CRISPR technologies promises to advance our understanding of gene regulatory networks, paving the way for novel therapeutic strategies.

## CHALLENGES IN SINGLE-CELL CRISPR ANALYSIS

Despite the remarkable progress in single-cell CRISPR technologies, there remains a need to extend perturbations to a genome-wide scale for comprehensive genetic target coverage. Achieving this requires addressing both technological and analytical challenges.

From a technological perspective, increasing cell throughput for individual perturbations is a major challenge for accurately assessing phenotypic changes. Its necessity is illustrated by a study that analyzed over 10,000 perturbations across 2.5 million cells (Replogle et al., 2022). This research utilized single-cell CRISPR technologies to create a comprehensive and multidimensional gene-phenotype map that provides insights into gene function and cellular behavior. By employing CRISPR interference screening to analyze the transcriptomes of over 100 cells per perturbation, the study identified novel regulators involved in composite phenotypes, including ribosome biogenesis, transcription, mitochondrial respiration, aneuploidy, and stress-specific regulation of the mitochondrial genome. While this study represents a significant achievement in genome–scale single-cell perturbation screening, it required over 300 sequencing lanes. To expand these screenings to in vivo or primary cell analysis, it is essential to implement ultra–high-throughput technologies that have been successfully demonstrated in other modalities. Current ultra–high-throughput technologies employ either plate-based combinatorial indexing, as seen in CRISPR-sciATAC (Liscovitch-Brauer et al., 2021), or initial indexing through in situ cDNA synthesis followed by incorporation into droplet-based systems. A key technical focus will be integrating these indexing strategies with sgRNA sequencing to analyze individual perturbations.

In the context of single-cell CRISPR screening data analysis, there are 2 primary objectives: (1) systematically elucidating regulatory circuits to identify novel regulators of specific phenotypes, and (2) modeling the effects of genetic perturbations to predict the outcomes of untested perturbations.

Single-cell CRISPR screens with multiple readouts offer more than a mere aggregation of individual marker analyses at the single-cell level. Although pseudobulk-based differential expression tests are a straightforward approach for analyzing a small number of perturbations simultaneously, they lack statistical robustness in single-cell data (Barry et al., 2024, Lee and Han, 2024). For sgRNA enrichment for individual markers at single-cell resolution, a rank-based test statistic provides a more robust assessment (Yang et al., 2020).

Nevertheless, as the scale of single-cell CRISPR screens increases, the framework for simultaneously quantifying regulatory relationships between multiple target genes and markers becomes crucial (Peidli et al., 2024). Regression-based approaches are widely employed in this context, utilizing a single-cell guide assignment matrix as a covariate and single-cell molecular readouts as responses to simultaneously map the effects of multiple target guides on various markers (Dixit et al., 2016, Frangieh et al., 2021, Yang et al., 2020, Zhou et al., 2023). However, permutation tests, which are commonly used to generate null distributions to calculate statistical significance, introduce a significant computational bottleneck due to their linearly scaling time complexity with the number of sgRNAs. Despite efforts to reduce computation time (Barry et al., 2024, Frangieh et al., 2021), permutation-based significance tests for genome–scale target guides remain computationally expensive.

Aside from the computational time constraints, the increased number of target guides also results in high sparsity in the covariate matrix, which can lead to insufficient estimation of effect sizes when using simple linear regression. One approach addressing this issue is Gaussian Sparse Factor Analysis (GSFA), a Bayesian factor analysis framework (Zhou et al., 2023). GSFA densifies the sparse covariate matrix by jointly learning a linear weight matrix and a sparse loading matrix, which map target guides to factors and factors to markers, respectively. Unlike permutation tests, GSFA uses Bayesian methods to assess significance, providing a more sensitive identification of regulatory relationships. However, the Bayesian end-to-end learning process of weight matrices in GSFA limits its scalability, making it feasible primarily for hundreds of guides. Therefore, there is room for improvement in regression-based approaches to address both computational scalability and statistical robustness as the number of target guides increases in single-cell CRISPR screens. Prediction of the effects of unseen perturbations primarily relies on generative models, which learn latent representations for each cell (Bereket and Information, T. K.-A. in N., & 2024, undefined, 2024, Lopez et al., 2018, Piran et al., 2024, Roohani et al., 2024, Tu et al., 2024, Weinberger et al., 2023). Models such as scVI (Lopez et al., 2018) naturally capture these latent representations, encoding information about the original cell profiles, thus are widely used for dimensionality reduction. In the context of single-cell CRISPR screens, latent representations of individual cells can be aggregated by target gene to estimate the effects of individual perturbations. Additionally, by manipulating these learned latent representations, it is possible to generate counterfactual observations and predict the effects of previously untested perturbations.

Recent advancements in generative models mainly focus on the interpretability or disentanglement of latent representations. Specifically, interpretability is enhanced by refining these representations to be sparse, which makes them aligned with annotated biological processes (Bereket and Information, T. K.-A. in N., & 2024, undefined, 2024, Lopez et al., 2022). Efforts are also being made to disentangle perturbation-specific responses (or salient representations) from shared background representations (Weinberger et al., 2023), and incorporation of additional supervised guidance of salient representation is explored in single-cell CRISPR screens (Tu et al., 2024).

Noisy observations also pose a significant challenge in modeling molecular readouts because perturbation readouts are influenced by stochastic factors, such as limited CRISPR system efficacy and sequencing dropouts. While the variable efficacy has been addressed in pooled CRISPR screens, this concern in single-cell CRISPR screens has not been thoroughly explored (Morgens et al., 2016). Given that CRISPR perturbations often produce weak signals in molecular readouts, comprehensive benchmarking of representation learning methods—covering aspects such as interpretability, disentanglement, and sensitivity—is crucial. Nonetheless, this remains challenging due to the relatively small number of genetic perturbations in typical experiments and the scarcity of genome–scale single-cell CRISPR screen data (Peidli et al., 2024).

## CONCLUSION

The evolution of single-cell technologies, which began with transcriptomics, has expanded to encompass genome, epigenome, and protein analysis, as well as immune profiling and genetic perturbation. More recently, innovations have significantly enhanced scalability in sample and cell numbers, achieved through the development of various demultiplexing approaches and combinatorial indexing methods. By obtaining multilayered information at the single-cell level from heterogeneous cell populations present in biological samples, these advancements have enabled the derivation of unique biological insights and facilitated a more holistic interpretation of cellular behavior, surpassing what could be achieved through the mere combination of single-omics data.

Although achieving genome-wide scale is crucial for single-cell CRISPR screening technologies, the necessary ultra–high-throughput technology to support this has yet to be developed. Furthermore, from an analytical perspective, analysis methods should be able to handle inherently complex and noisy observations effectively and efficiently, in line with the growing scale of experimental technologies.

In conclusion, the rapid development and integration of multiplexed, multimodal single-cell technologies have profoundly impacted our understanding of cellular biology. From observational studies to perturbation analyses, these technologies offer unprecedented precision and depth, paving the way for novel therapeutic strategies and personalized medicine. As we continue to address existing challenges and push the boundaries of single-cell analysis, the future holds immense potential for further discoveries and applications in biomedical research.

## Author Contributions

Junha Park: Writing—review and editing, Writing—original draft. Byungjin Hwang: Writing—review and editing, Writing—original draft, Conceptualization. Su-Hyeon Lee: Writing—review and editing, Writing—original draft.

## Declaration of Competing Interests

This review adheres to all ethical standards and declares that there are no conflicts of interest related to this work.
